# Periodontal Diseases Knowledge, Attitudes and Practices of Patients With Hypertension: A Cross-Sectional Study in Two Hospitals in the West Region (Cameroon)

**DOI:** 10.1155/ijod/1749412

**Published:** 2025-06-19

**Authors:** Pascaline Magne Tambe, Sylvain Raoul Simeni Njonnou, Christian Ngongang Ouankou, Christian Deube Ngako, Fernando Kemta Lekpa, Clarisse Mapa-Tassou, Herna Stella Chimy Tchounchui, Siméon Pierre Choukem, Charles Muhima Pilipili

**Affiliations:** ^1^Dental School, Higher Institute of Health Sciences, Université des Montagnes, Bangangté, Cameroon; ^2^Department of Internal Medicine and Specialities, Faculty of Medicine and Pharmaceutical Sciences, University of Dschang, Dschang, Cameroon; ^3^Department of Internal Medicine, Dschang Regional Annex Hospital, Dschang, Cameroon; ^4^Department of Internal Medicine, Health and Human Development (2HD) Research Network, Douala, Cameroon; ^5^Department of Internal Medicine, Yaounde Teaching Hospital, Yaounde, Cameroon; ^6^Department of Epidemiology and Public Health, Faculty of Medicine and Pharmaceutical Sciences, University of Dschang, Dschang, Cameroon; ^7^Department of Internal Medicine, Douala General Hospital, Douala, Cameroon; ^8^Ministry of Public Health, Yaounde, Cameroon

**Keywords:** attitudes, cameroon, hypertension, knowledge, oral health, periodontal diseases, practices

## Abstract

**Background:** Hypertension affects a large proportion of the world's population. There is a bidirectional association between hypertension and periodontal disease (PD). This study aimed to assess the oral health knowledge, attitudes and practices (KAP) of patients living with hypertension followed up at the Bafoussam Regional Hospital (BRH) and the Dschang Regional Annex Hospital (DRAH).

**Methods:** This cross-sectional study was conducted from February to April 2024 in two tertiary health facilities of the west Cameroon region, targeting hypertensive outpatients or followed up in cardiology and internal medicine who meet the inclusion criteria. Consecutive sampling was used to collect sociodemographic data, past history, and oral health KAP via a face-to-face questionnaire and medical records. The KAP were analyzed using the Essi and Njoya grid. Logistic regression was used to determine the factors associated with poor KAP (*p*  < 0.05).

**Results:** A total of 367 people (143 males) with a median age of 62 years (IQR 53-70) were recruited. The median duration of hypertension was 5 years (IQR 2-10). Almost all participants (91.8%) had poor knowledge, 58.6% had harmful attitudes and 67.6% had inadequate practices. Living in an urban area (aOR: 0.43 [95% CI 0.19-0.97]; *p*=0.042) was negatively associated with poor knowledge. Recently diagnosed hypertension (aOR: 1.74 [95% CI 1.11-2.76]; *p*=0.015) and poor knowledge (aOR: 3.50 [95% CI 1.55-7.92]; *p*=0.003) were positively associated with harmful attitudes. Primary education (aOR: 1.32 [95% CI 1.05-2.676]; *p*=0.042) and low monthly income (aOR: 1.50 [95% CI 1.02-3.14]; *p*=0.046) were positively associated with harmful practices.

**Conclusion:** Most participants had insufficient knowledge of oral health and adopted harmful practices. Highlighting the need for carers and decision-makers to raise awareness and educate hypertensive patients about the relationship between their disease and oral health using the associated factors identified.

## 1. Introduction

Hypertension is defined as a systolic blood pressure (SBP) of 140 mmHg and/or diastolic blood pressure (DBP) of 90 mmHg [1]. According to a World Health Organisation (WHO) report published in 2021, cardiovascular disease (CVD) is responsible for around 210.5 million deaths worldwide, or almost a third of all deaths. Of these deaths, 9.4 million are attributable to complications of hypertension, according to the WHO in 2013 [2]. Almost one-third (32%) of the world's adult population is hypertensive but this prevalence is expected to decrease to 20.3% in 2024 [3, 4]. According to the WHO, average blood pressure is higher in Africa than in Europe and the United States [5]. Hypertension is thought to affect 25% –40.2% of the adult population in sub-Saharan Africa (SSA), with high rates in urban areas. This prevalence is increasing among women. In Mali, one adult woman in three is hypertensive, and the prevalence of metabolic risk factors for hypertension has been estimated at 34.7% or (34% of men and 35% of women) [6]. In Cameroon, the prevalence of hypertension in rural areas undergoing urbanization has more than doubled in the space of 10 years, due to factors, such as increasing obesity and alcohol consumption [2]. A recent study in Dschang, a semirural city, found a prevalence of 57.6% among community dwellers [7]. However, 30.9% of the Cameroonian population is hypertensive and only 8.8% of them have their blood pressure controlled [8]. Not only does hypertension have a high morbidity and mortality rate, but it also consumes a great deal of medical and social resources and places a heavy economic burden on families and society [2, 9]. Uncontrolled hypertension is associated with disabling lesions leading to heart disease, stroke, and kidney disease [10]. It also impacts the oral cavity, increasing the risk of periodontal disease (PD) [11, 12].

Periodontal diseases (PDs), namely gingivitis and periodontitis, are chronic inflammatory diseases that affect the supporting tissues of the teeth, caused by a long-term accumulation of dental biofilm and tartar. PD is one of the most prevalent oral diseases in the world [13]. Its prevalence varies from 20% to 50% in the general population in Cameroon [14]. In Africa, the distribution of PD varies from one region to another. In the Menabe region of Madagascar it reaches 45%, while it is 33% in Ghana, 27.5% in Nigeria, and 30% in Senegal [15, 16]. In Cameroon, the general prevalence is 15% [17], almost half (46%) of Cameroonian hypertensive patients in Douala present a PD [18]. Another Cameroonian study conducted in 2018 in patients with CVD found a prevalence of 62.2% for gingivitis and 15% for periodontitis, with periodontitis associated with CVD [19]. Several local, regional, and worldwide studies demonstrate an association between hypertension and PD [11, 12, 18, 20]. Some studies suggest that PD influences the control of hypertension as inflammatory mediators released in the local environment following periodontitis could enter the bloodstream and lead to chronic systemic inflammation [11]. Similarly, periodontitis is more common in people with uncontrolled hypertension than in those with controlled hypertension (35.5% versus 9.5%) [18]. This therapeutic approach could be explored in uncontrolled hypertensive patients.

However, a recent study conducted, found that patients with CVD had an overall moderate level of knowledge and attitudes, but their oral health practices were below average [16]. In our context, patients living with hypertension do not benefit from an oral disease prevention program. Public health initiatives aimed at educating and raising the awareness of this target group will make it possible to promote oral health and prevent oral pathologies, thereby helping to improve their quality of life. It is in this context that we proposed to assess the knowledge, attitudes, and oral health practices of patients living with hypertension followed up in two hospitals in the west region of Cameroon.

## 2. Methods

### 2.1. Study Design and Setting

This cross-sectional study targeted patients with hypertension attending outpatient consultations or follow-ups at the Bafoussam Regional Hospital (BRH) and the Dschang Regional Annex Hospital (DRAH). As Regional Hospitals, these two health facilities play a central role in the provision of quality health care for the population of the west Cameroon Region, which justifies our choice.

The BRH is located in Bafoussam, the capital of Cameroon's west region. It is a third-category public health facility (of the three in the west region) in Cameroon's health pyramid. Two cardiologists carry out outpatient consultations and follow-ups of patients with hypertension in the cardiology department.

The DRAH is also a third-category public health facility located in the Dschang district (45 km from Bafoussam), opposite the University of Dschang. Outpatient consultations and follow-ups of patients with hypertension are carried out in the internal medicine department by a cardiologist and an internist.

### 2.2. Study Population

Our study population consisted of all outpatients living with hypertension coming for consultation or follow-up to the Cardiology and Internal Medicine department of the BRH and DRHA from 1^st^ February 2024–30^th^ April 2024 (3 months). All those who gave informed consent during the study period were included. All patients living with hypertension with a diagnosis of less than 1 year old were excluded from the study. The minimum sample size was calculated using the prevalence (30.9%) of hypertension in Cameroon [5], with a margin of error of 5% (*Z* = 1.96). The minimum sample size was 328 participants.

### 2.3. Data Collection

Data were collected after explaining the purpose of the study to the patient and obtaining their signed informed consent. We administered a preestablished and pretested face-to-face questionnaire to collect the following data: (i) sociodemographic characteristics (age, sex, marital status, level of education, monthly income, area of residence, and profession); (ii) knowledge (10 questions); (iii) attitudes (9 questions); and (iv) practices (10 questions). We also used patients' medical records to collect information on hypertension (grade, duration of diagnosis, control, and treatment) and comorbidities.

### 2.4. Operational Definitions

• Hypertensive patient: Any person with a documented diagnosis of hypertension or taking antihypertensive treatment.
• Controlled hypertensive: Any hypertensive patient with a SBP of less than 140 mmHg and a DBP of less than 90 mmHg on the day of the survey.
• Grade of hypertension: Grade I: SBP: 140–159 and/or DBP 90–99 mmHg; Grade II: SBP 160–179 mmHg and/or DBP 100–109 mmHg; and Grade III: SBP ≥ 180 mmHg and/or DBP ≥ 110 mmHg.
• Duration of diagnosis of hypertension: Recent (<5 years) and old (≥5 years).
• Body weight status: Was defined as follows: Normal weight (18.50 ≤ BMI ≤ 24.99 kg/m^2^); overweight BMI (25 ≤ BMI ≤ 29.99 kg/m^2^); and obese (BMI ≥ 30 kg/m^2^).
• Physical activity: Was defined as the presence of at least three walking episodes of 45 min in a week.
• Monthly income: Low (<USD 86.3), medium (USD 86.3 and 258.9), and high (> USD 258.9) [21].
• Diabetic patient: Any person with a documented diagnosis of diabetes or taking antidiabetic treatment.
• Tobacco consumption: Subject declaring smoking or having stopped smoking tobacco less than 6 months ago.

### 2.5. Statistical Analysis

The data were collected using the koboCollect application and analyzed using Statistical Package for the Social Sciences 20 (SPSS, IBM Corp, Armonk, NY, version 20) software. Quantitative variables were expressed as median and interquartile range after checking the distributisson. Qualitative variables were expressed in terms of numbers and percentages.

To assess the different items in the knowledge, attitudes and practices (KAP), we used the Essi and Njoya [22] grid. It is subdivided, into knowledge as, poor (score < 25% of correct answers), insufficient (score = [25%–50%] of correct answers), average (score = [50%–70%] of correct answers), and good (score ≥ 70% of correct answers). Attitudes were: harmful (score < 25% of correct answers), wrong (score = [25%–50%] of correct answers), approximate (score = [50%–70%] of correct answers), and right (score ≥ 70% of correct answers). Practices were harmful (score < 50% of correct responses), inadequate (score = [50%–70%] of correct responses), and adequate (score ≥ 70% of correct responses).

The chi-square test and Fisher's exact test were used to compare proportions in bivariate analysis. The comparison groups were: poor knowledge versus other levels of knowledge; harmful attitudes versus other levels of attitudes; harmful and inadequate practices versus adequate practices.

Multivariate analysis with calculation of adjusted odds ratios and 95% confidence intervals was used to determine the factors associated with poor knowledge, harmful attitudes, and practices. We included in the multivariate model the variables with *p* < 0.05 in the bivariate analysis. Differences were considered statistically significant for values of *p* < 0.05.

### 2.6. Ethical considerations

We obtained ethical clearance (N°2024/073/UdM/PR/CEAQ) from the Ethics and Quality Assurance Committee of the Université des Montagnes and research authorizations from the Directors of the BRH and the DRAH. Informed consent was obtained from each participant. The questionnaire respected the rules of anonymity and the information collected during our study was kept confidential. The data and results were used for purely scientific purposes. This research was conducted in accordance with the Helsinki principles.

## 3. Results

### 3.1. General Characteristics

During the data collection period, we approached 387 hypertensive patients. Of these, 8 refused to participate and 12 were excluded because they had been diagnosed with hypertension less than a year previously. In the end, 367 patients were retained.

The median age of the participants was 62 years (IQR 53–70) with extremes ranging from 30 to 90 years. Women accounted for 61.0% compared with 39.0% of men. Nearly half of the participants (48.5%) had a primary education. More than half (54.8%) had a low monthly income (<USD 86.3). Less than half (42.5%) lived in an urban area (Table 1). More than a third (35.7%) of patients had grade II hypertension, compared with 26.2% for grade I. The median duration of diagnosis of hypertension was 5 years (IQR 2–10), with a minimum of 1 year and a maximum of 40 years. More than half (54.8%) of the participants had an old diagnosis of hypertension. The majority (76.0%) of patients had controlled hypertension. The most common comorbidity was diabetes (24.5%), and less than a quarter (23.4%) of the participants were of normal weight (Table 2).

### 3.2. Knowledge

In terms of participants' knowledge, we found that 99.2% had not received any information about the link between hypertension and oral health. The majority (97.5%) did not know that hypertension increases the risk of developing PD. We also note that only a minority (2.5%) of patients knew that an oral problem could affect the control of hypertension. Of the participants, 98.6% did not realize that treating bleeding gums can improve hypertension control. More than a third (35.1%) knew the recommended brushing time but only 4.9% knew the brushing period. A minority (4.1%) of patients knew the recommended annual frequency of dental checkups. Overall, 91.8% (*n* = 337) of participants had poor knowledge, 7.1% (*n* = 26) insufficient knowledge, 0.5% (*n* = 2) average knowledge, and 0.5% (*n* = 2) good knowledge (Table 3).

### 3.3. Attitudes

In terms of attitudes, almost two-thirds (62.1%) of hypertensive patients had never consulted a dentist, compared with 28.6% who had done so but more than 2 years previously. Only 3.8% of patients thought that a periodontal problem could be associated with their general state of health. Furthermore, 89.4% did not feel the need to inform the dentist about their hypertension status. The majority (85.0%) of patients stated that they are willing to obtain more information about their risk of PD. Dental pain (98.4%) was the main reason that would compel patients to make a dental visit, compared with 7.9% for a routine visit. The majority (98.4%) of participants had not received a referral from their doctor for a dental consultation. Overall, 58.6% (*n* = 215) had harmful attitudes, 34.9% (*n* = 128) incorrect attitudes, 4.6% (*n* = 17) approximate attitudes, and 1.9% (*n* = 7) correct attitudes (Table 4).

### 3.4. Practices

About practices, almost all (99.2%) of the participants brushed their teeth and almost three-quarters (74.5%) of them used a toothbrush and toothpaste. More than half (52.2%) of patients cleaned their teeth once a day. A large majority (83.5%) said they used a soft-bristled toothbrush. Only 8% of participants brushed in the morning after eating and in the evening at bedtime. The majority (90.5%) did not use dental floss, although 73.3% used a toothpick. Overall, 67.6% (*n* = 248) of participants had inadequate practices, 15.8% (*n* = 58) had harmful practices and 16.6% (*n* = 61) had adequate practices (Table 5).

### 3.5. Factors Associated With Poor Knowledge, Harmful Attitudes and Practices

#### 3.5.1. Factors Associated With Poor Knowledge

In bivariate analysis, age <40 years (cOR = 0.24 [95% CI 0.07–0.79]; *p*=0.003), university level of education (cOR = 0.24 [95% CI 0.11–0.54]; *p*=0.001), living in an urban area (cOR = 0.39 [95% CI 0.18–0.96]; *p*=0.016), teaching profession (cOR = 0.22 [95% CI 0.08–0.57]; *p*=0.004), physical activity (cOR = 0.28 [95% CI 0.09–0.82]; *p*=0.003) and the presence of eye disease (cOR = 0.19 [95% CI 0.06–0.68]; *p*=0.020) were negatively associated with poor knowledge, whereas primary school level (cOR = 2.79 [95% CI 1.21–6.46]; *p*=0.013) was positively associated with poor knowledge.

After multivariate analysis, the factors significantly associated with poor knowledge were living in an urban area (aOR = 0.43 [95% CI 0.19–0.97]; *p*=0.042) and the presence of eye disease (aOR = 0.21 [95% CI 0.060–0.79]; *p*=0.021) (Table 6).

#### 3.5.2. Factors Associated With Harmful Attitudes

We found in bivariate analysis that age ≥80 years (cOR = 2.26 [95% CI 1.99–5.18]; *p*=0.048), low monthly income (cOR = 1.59 [95% CI 1.047–2.420]; *p*=0.029), living in an rural area (cOR = 1.68 [95% CI 1.02–2.77]; *p*=0.042), a recent diagnosis of hypertension (cOR = 1.71 [95% CI 1.12–2.62]; *p*=0.012), not taking antihypertensive treatment (cOR = 2.32 [95% CI 1.13–4.76]; *p*=0.018), poor knowledge (cOR = 3.67 [95% CI 1.63–8.26]; *p*=0.001) were positively associated with harmful attitudes. In contrast, university level of education (cOR = 0.42 [95% CI 0.23–0.77]; *p*=0.004), living in an urban area (cOR = 0.65 [95% CI 0.43–0.99]; *p*=0.044), having controlled hypertension (cOR = 0.58 [95% CI 0.35–0.97]; *p*=0.036), the presence of heart failure (cOR = 0.29 [95% CI 0.11–0.71]; *p*=0.005), and obesity (cOR = 0.57 [95% CI 0.37–0.87]; *p*=0.009) were negatively associated with harmful practices.

Finally, multivariate analysis showed that the factors significantly associated with harmful attitudes were: a recent diagnosis of hypertension (aOR = 1.74 [95% CI 1.12-2.74]; *p*=0.015), poor knowledge (aOR = 3.50 [95% CI 1.55-7.92]; *p*=0.003), the presence of heart failure (aOR = 0.23 [95% CI 0.09-0.61]; *p*=0.003) and obesity (aOR = 0.62 [95% CI 0.40-0.97]; *p*=0.035) (Table 7).

#### 3.5.3. Factors Associated With Harmful Practices

Bivariate analysis showed that primary education (cOR = 2.62 [95% CI 1.45-4.74]; *p*=0.001), low monthly income (cOR = 3.24 [95% CI 1.80-5.84]; *p* < 0.001), living in a rural area (cOR = 2.3 [95% CI 1.11-5.2]; *p*=0.023), being housewife (cOR = 3.05 [CI 95% 1.06-8.77]; *p*=0.030), poor knowledge (cOR = 3.32 [CI 95% 1.49-7.40]; *p*=0.008), and harmful attitudes (cOR = 2.18 [CI 95% 1.25-380]; *p*=0.006) were positively associated with harmful practices. However, age <40 years (cOR = 0.18 [CI 95% [0.06-0.49]; *p*=0.002), single status (cOR = 0.28 [CI 95% 0.09-0.81]; *p*=0.024), university level of education (cOR = 0.24 [CI 95% 0.13-0.46]; *p* < 0.001), high monthly income (cOR = 0.21[CI 95% 0.10-0.45]; *p* < 0.001), living in an urban area (cOR = 0.57 [95% CI 0.33-0.99]; *p*=0.045), being a teacher (cOR = 0.23 [95% CI 0.10-0.51]; *p* < 0.001), and physical activity (cOR = 0.28 [95% CI 0.11-0.67]; *p*=0.007) were negatively associated with harmful practices.

Finally, multivariate analysis revealed that the factors significantly associated with harmful practices were: primary education (aOR = 1.32 [95% CI 1.05-2.68]; *p*=0.042), low monthly income (aOR = 1.50 [95% CI 1.02-3.14]; *p*=0.046), poor knowledge (aOR = 2.77 [CI 95% 1.22-6.281]; *p*=0.015), harmful attitudes (aOR = 1.95 [CI 95% 1.10-3.44]; *p*=0.022), and university level of education (aOR = 0.70 [CI 95% 0.27-0.99]; *p*=0.028) (Table 8).

## 4. Discussion

Hypertension remains one of the most serious issues in SSA. Its prevalence is high, few patients are correctly treated and achieve control. Recent data suggest an association between PDs, particularly periodontitis and hypertension. This might be a suitable therapeutic approach. This cross-sectional study, on 367 people living with hypertension followed in two regional hospitals of the west region, aimed to assess their knowledge, attitudes, and oral health practices. Almost all participants (91.8%) had poor knowledge, 58.6% had harmful attitudes, and 67.6% had inadequate practices. Living in an urban area was negatively associated with poor knowledge. Recently diagnosed hypertension and poor knowledge were positively associated with harmful attitudes. Primary education and low monthly income were positively associated with harmful practices.

In this study, less than 1% of participants had received information on the relationship between hypertension and oral health, while 99.2% had not. This could be due to a lack of knowledge or information about the relationship between hypertension and oral health. These results show that there is an urgent need for healthcare staff to raise awareness among people living with hypertension [23].

Only 2.2% of patients were aware that gingival bleeding can affect hypertension. These results are much lower than those found in 2015 in Iran (40.0%) [24]. This could be explained by the fact that the participant's level of education was high in that study (university studies) in contrast to ours, where the participant's level of education was low (primary).

Of these participants, 98.6% were unaware that treating bleeding gums could improve blood pressure, and 97.5% were unaware that hypertension increases the risk of developing gum problems. These results may reflect the fact that very few patients receive oral health information from their general practitioner (GP). Their GP should also provide a brief oral health overview to raise awareness of oral health issues among these patients.

Similarly, the majority (95.9%) thought they would go to the dentist if they had a problem. Our results are higher than those in Australia (2019), where 28.0% only were going 28.0% to the dentist in this case [25]. On the one hand, this difference could be explained by the fact that our study population was predominantly primary-educated, as opposed to the higher educational level in Sanchez's study. On the other hand, it could be explained by the fact that Australia has better social health coverage than Cameroon, which does not. To overcome this problem, health services need accurate information on the oral health status of population groups, particularly patients with chronic pathologies.

Overall, the majority (91.8%) of participants had poor knowledge. Our results disagree with those of Saudi Arabia in 2020, where 47% of participants had good knowledge [26]. This could be explained by the fact that most of the knowledge received comes from personal experience and that of relatives. The relatively low level of schooling does not allow them to gather reliable information. But it's also because healthcare staff don't take enough time to explain the relationship between their pathology and oral health. It is therefore, essential to educate patients living with hypertension about their increased risk of oral health problems, motivate them to adopt good oral health behaviors and facilitate access to dental care.

The interest in oral health was very low among this population. This was confirmed by several points. First, almost two-thirds (62.1%) of participants had never had a dental consultation. The present study shows a difference from a study in 2012 in Madagascar, where 79.2% had benefited from a dental consultation [16]. This may be linked either to the fact that most participants' monthly income was low or that their hypertension was already a financial burden, and the lack of advice given by their GP. Second, the participant's main reason for making a dental consultation was pain, that is 98.4% of cases. These results are superior to those found in Mali, where the main reason for consultation was pain at 78.22% [27]. This could reflect the fact that most patients do not have the culture of going to the dentist for routine consultations. It could also be due to a lack of financial resources. Public authorities need to think about integrating dental consultations into consultations for patients living with hypertension, to facilitate their access to dental care. Third, most participants (89.4%) did not feel the need to inform the dentist about their state of health. These results are higher than those in Saudi Arabia (29%) [26]. These results could be explained by the fact that patients have not been made sufficiently aware of the importance of stating their history every time they come face to face with healthcare personnel. Finally, almost all (98.1%) had not received oral health advice from their GP. These results are at odds with those of the USA, where the majority of doctors provided oral health advice to patients because they thought it wiser to take more precautions by sending them to the dentist for better management [28]. This difference could be explained by the fact that, in developed countries, the management of pathology is global, unlike in developing countries. These results could also be justified by the fact that the majority of healthcare staff focus solely on the reason for consultation that prompts patients to consult, and this could be a problem of medical culture [23].

Globally, the oral hygiene of this population was low: a little more than half (52.2%) of the participants cleaned their teeth once a day while 43.4% brushed in the morning only before meals. These results are similar to those of another study in Mali, which found that 41.8% cleaned their teeth once a day mainly the morning before meals [29]. This could be justified by the fact that these patients do not master the frequency of brushing or that they considered morning brushing to be more important, in effect a cultural problem. Hence, there is a need to educate them about oral hygiene. Similarly, only a minority (9.5%) used dental floss and 1.1% used interdental humps, with most using toothpicks once the food had become trapped in the interdental spaces. These results are at odds with those found in 2021a study in Nigeria, where 85.5% of participants used dental floss and mouthwash [30]. This could be due to patients' poverty or lack of knowledge about how to use them.

Overall, 67.6% of participants had inadequate practices. These results are at odds with those of Nigeria, who found that participants had better practices.

The multivariate analysis showed that living in an urban area reduced the risk of having the wrong knowledge. This could be justified by the fact that living in an urban area exposes people to good knowledge, since in urban areas people find it easier to be informed about oral health through several available information channels, which is still not the case in rural areas [31].

We found a positive association between a recent diagnosis of hypertension, poor knowledge, and harmful attitudes. This could be explained, on the one hand, by the fact that subjects living with recently diagnosed hypertension probably do not yet have complications, are not well-controlled and would therefore, be less vigilant about taking care of their health, particularly their oral health [12]. On the other hand, they are not yet sufficiently informed about their disease, its complications, and its relationship with oral health [12, 24].

Heart failure and obesity have been identified as protective factors associated with harmful attitudes. As heart failure is a complication of hypertension, patients are more aware of the risks associated with their multiple comorbidities. Obesity itself is associated with a high risk of PD [17, 32, 33]. It is therefore, possible that obese hypertensive subjects have already experienced or are more aware of this risk and therefore, adopt less harmful attitudes towards their oral health.

This study also showed a positive association between primary education, low monthly income, poor knowledge, harmful attitudes, and harmful practices. On the other hand, higher education was negatively associated with harmful practices. Hypertensive people with only primary education may have less access to information and understanding of good oral hygiene practices and the importance of maintaining good oral health than those with a university education. The level of education is therefore, a decisive indicator of the education of these patients. Hypertensive patients with low monthly incomes may not be able to afford basic quality oral hygiene products, especially as the few resources they have are likely to be directed towards the management of their disease. This can lead to less effective oral hygiene and therefore, a greater risk of PD. This result suggests that universal health coverage is a reality for these patients, enabling them to bear certain medical costs. The positive association we found between poor knowledge, harmful attitudes, and harmful practices is a logical result. If people don't know about good oral hygiene practices, they won't be able to adopt them. What's more, a negative or indifferent attitude towards oral health can reduce motivation to adopt healthy practices, such as brushing regularly and using dental floss instead of a toothpick.

Although this study did not specifically address the impact of culture, traditions and beliefs on oral hygiene and particularly PD, it must be recognized that in many cultures, specific practices like using certain traditional remedies or specific methods of cleaning teeth may be the norm, potentially influencing the effectiveness of oral hygiene, and the development of PD. Furthermore, beliefs about the severity of PD and the effectiveness of dental treatments may negatively influence treatment-seeking behavior [34]. Some cultures may perceive gum disease as, less severe or have, different expectations of treatment outcomes, resulting in delayed or inadequate care [35]. Nevertheless, studies have shown an association between PD and hypertension, suggesting that periodontal treatment may improve blood pressure control. Cultural beliefs and practices may influence the way people with hypertension manage their oral health and have a potential impact on their general health. To improve oral health and manage hypertension, it is essential to adapt health interventions to the specific cultural context. This includes taking into account cultural norms, beliefs, and practices related to oral health and hypertension [36].

## 5. Study Limitations

This study is the first in Cameroon to assess the KAP of hypertensive patients on oral health. It took into account populations living in urban and rural areas. Nevertheless, we admit that it has certain limitations, particularly the fact that it is hospital-based and limited to two hospitals. The non-probability sampling method did not give all hypertensive patients an equal probability to participate. We did not explore the impact of culture (beliefs and traditional practices) on oral health KAP and we did not perform an oral examination) of these patients. The results of this study cannot, therefore, be generalized without caution.

## 6. Conclusion

Hypertensive patients are unaware that their disease can influence their oral health and vise versa. Overall, they have poor knowledge of oral health, negative attitudes, and inadequate practices. The area of residence influences the level of knowledge, the duration of hypertension influences attitudes, level of education, and monthly income influence practices. These results highlighting the need for carers, decision-makers to raise awareness, and educate hypertensive patients about the relationship between their disease and oral health using the associated factors identified.

### 6.1. What is Known on the Topic/What This Study Adds

What is known on the topic?

• Hypertension affects a large proportion of the world's population.
• There is a bidirectional relationship between hypertension and PD.

What this study adds:

• Hypertensive patients need education on the relationship between their disease and oral health.
• Hypertensive patients living in urban areas have more information about oral health than those living in rural areas.
• Newly diagnosed hypertensives have more negative oral health attitudes.
  Hypertensive patients with primary education and low monthly income have more negative oral health practices.

## Figures and Tables

**Table 1:** Distribution of sociodemographic characteristics of the study population.

Variables	Terms	*n*	%
Age (years)	<40	16	4.4
	40–60	132	36.0
	60–80	187	51.0
	≥80	32	8.7

Sex	Male	143	39.0
	Female	224	61.0

Marital status	Married	253	68.9
	Single	15	4.1
	Divorced	8	2.2
	Widowed	91	24.8

Level of education	None	49	13.4
	Primary	178	48.5
	Secondary	88	24.0
	University	52	14.2

Monthly income (USD)	Low	201	54.8
	Average	131	35.7
	High	35	9.5

Residence area	Urban	156	42.5
	Semi-urban	121	33.0
	Rural	90	24.5

Occupation	Retailer	61	16.6
	Farmer	69	18.8
	Unemployed	48	13.1
	Teacher	28	7.6
	Housewife	58	15.9
	Retired	75	20.4
	Others	28	7.6

**Table 2:** Description of hypertension characteristics and comorbidities.

Variables	Terms	*n*	%
Hypertension grade	Grade I	96	26.2
	Grade II	131	35.7
	Grade III	68	18.5
	Not classified	72	19.6

Duration of hypertenion diagnosis	Recently	166	45.2
	Old	201	54.8

Controlled hypertension	Yes	279	76.0
	No	88	24.0

Hypertension treatment category	Monotherapy	136	37.1
	Bitherapy	144	39.2
	Tritherapy	31	8.4
	Quadritherapy	12	3.3
	None	44	12.0

Tobacco consumption	Yes	11	3.0
	No	356	97.0

Physical activities	Yes	23	6.3
	No	344	93.7

Comorbidities	Diabetes	90	24.5
	Gout	70	19.1
	Heart failure	23	6.3
	Eye disease	14	3.8

Weight status (BMI)	Normal	86	23.4
	Overweight	120	32.7
	Obese	161	43.9

**Table 3:** Hypertensive patients' knowledge of oral health.

Variables	Yes, *n* (%)	Do not know, *n* (%)
Already have received information on the relationship between hypertension and oral health	3 (0.8)	364 (99.2)
There is a link between hypertension and oral health	2 (0.5)	365 (99.5)
Bleeding gums can affect hypertension	8 (2.2)	359 (97.8)
Hypertension can affect the gums	7 (1.9)	360 (98.1)
Treatment of bleeding gums can improve blood pressure control	5 (1.4)	362 (98.6)
Hypertension increases the risk of developing gum disease	9 (2.5)	358 (97.5)
Oral diseases can have a negative impact on hypertension control	9 (2.5)	358 (97.5)
Knows the recommended frequency of annual dental checkups	15 (4.1)	352 (95.9)
Knows recommended brushing period	18 (4.9)	349 (95.1)
Knows recommended brushing duration	129 (35.1)	238 (64.9)

**Table 4:** Attitudes of hypertensive patients towards oral health.

Variables	Terms	*n*	%
Have already visited a dentist	Yes	139	37.9
	No	228	62.1

Date of last dental visit	<1 year	23	6.3
	1–2 years	11	3.0
	>2 years	105	28.6
	Never consulted	228	62.1

Thinks that a gum problem may be associated with his general state of health	Yes	14	3.8
	No	34	9.3
	No opinion	319	86.9

Feels the need to inform dentist of hypertensive condition	Yes	39	10.6
	No	328	89.4

Wants more information about their risk of gum disease	Yes	312	85.0
	No	55	15.0

Clarify hypertensive status prior to dental treatment	Yes	44	12.0
	No	88	24.0
	Sometimes	7	1.9
	Never consulted	228	62.1

Situations that would require you to consult a dentist	Tooth pain	361	98.4
	Tooth mobility	286	77.9
	Bleeding gums	259	70.6
	Bad breath	117	31.9
	Checkup	29	7.9

Your doctor has already given you advice on oral health	Yes	7	1.9
	No	360	98.1

Your doctor has already referred you to a dentist for a consultation	Yes	6	1.6
	No	361	98.4

**Table 5:** Participants' oral health prevention practices.

Variables	Terms	*n*	%
Tooth brushing	Yes	364	99.2
	No	3	0.8

Brushing materials (*n* = 364)	Toothbrush only	48	13.2
	Toothbrush and toothpaste	271	74.5
	Toothbrush and charcoal/ash	22	6.0
	Toothbrush and salt	7	1.9
	Toothbrush and bicarbonate	13	3.6
	Others	4	1.1

Toothbrush type (*n* = 364)	Soft	304	83.5
	Medium	46	12.6
	Hard	14	3.8

Daily brushing frequency (*n* = 364)	Once	190	52.2
	Twice	166	45.6
	More than 2 times	8	2.2

Brushing time	Morning before meal only	158	43.4
	Morning after meal only	16	4.4
	After dinner only	10	2.8
	In the morning after meals and in the evening at bedtime	29	8.0
	In the morning before meals and in the evening at bedtime	148	40.7

Brushing duration (in min)	<2	2	0.5
	≥2	362	99.5

Use dental floss	Yes	35	9.5
	No	332	90.5

Use interdental brushes	Yes	4	1.1
	No	363	98.9

Use a mouthwash	Yes	14	3.8
	No	353	96.2

Toothpick use	Yes	269	73.3
	No	98	26.7

**Table 6:** Factors associated with poor knowledge.

Variables	Terms	Poor knowledge		*p*	Crude OR (CI 95%)	Adjusted *p*	Adjusted OR (CI 95%)
		Yes, *n* (%)	No, *n* (%)				
Age (years)	<40	12 (3.6)	4 (13.3)	**0.033**	**0.240 (0.072–0.797)**	0.225	0.431 (0.111–1.678)
	40-60	125 (37.1)	7 (23.3)	0.132	1.937 (0.808–4.645)	—	—
	60-80	170 (50.4)	17 (56.7)	0.514	0.778 (0.387–1.653)	—	—
	≥80	30 (8.9)	2 (6.7)	1	1.368 (0.311–6.026)	—	—

Sex	Male	130 (38.6)	13 (43.3)	0.609	0.821 (0.386–1.747)	—	—
	Female	207 (61.4)	17 (56.7)	—	—	—	—

Marital status	Married	232 (68.8)	21 (70.0)	0.896	0.947 (0.419–2.138)	—	—
	Single	13 (3.9)	2 (6.7)	0.361	0.562 (0.121–2.615)	—	—
	Divorced	8 (2.4)	0	1	Not applicable	—	—
	Widowed	84 (24.9)	7 (23.3)	0.847	1.091 (0.452–2.634)	—	—

Level of education	None	46 (13.6)	3 (10.0)	0.781	1.423 (0.415–4.980)	—	—
	Primary	170 (50.4)	8 (26.7)	**0.013**	**2.799 (1.212–6.464)**	0.539	1.362 (0.508–3.647)
	Secondary	80 (23.7)	8 (26.7)	0.719	0.956 (0.367–1.997)	—	—
	University	41 (12.2)	11 (36.7)	**0.001**	**0.239 (0.106–0.538)**	0.186	0.481 (0.162–1.423)

Monthly income (USD)	Low	187 (55.5)	14 (46.7)	0.352	1.425 (0.674–3.012)	—	—
	Average	119 (35.3)	12 (40.0)	0.652	0.912 (0.334–2.491)	—	—
	High	31 (9.2)	4 (13.3)	0.511	0.658 (0.216–2.009)	—	—

Residence area	Urban	137 (40.7)	19 (63.3)	**0.016**	**0.397 (0.183–0.960)**	**0.042**	**0.430 (0.191–0.968)**
	Semi-urban	114 (33.8)	7 (23.3)	0.241	1.680 (0.700–4.032)	—	—
	Rural	86 (25.5)	4 (13.3)	0.137	2.227 (0.756–6.583)	—	—

Occupation	Retailer	58 (17.2)	3 (10.0)	0.444	1.871 (0.549–6.375)	—	—
	Farmer	65 (19.3)	4 (13.3)	0.424	1.553 (0.624–4.606)	—	—
	Unemployed	46 (13.6)	2 (6.7)	0.400	2.213 (0.510–9.605)	—	—
	Teacher	21 (6.2)	7 (23.3)	**0.004**	**0.218 (0.084–0.567)**	0.344	0.542 (0.153–1.924)
	Housewife	52 (15.4)	6 (20.0)	0.600	0.730 (0.284–1.872)	—	—
	Retired	69 (20.5)	6 (20.0)	0.951	1.030 (0.405–2.618)	—	—
	Others	26 (7.7)	2 (6.7)	1	1.170 (0.264–5.190)	—	—

Hypertension grade	Grade I	86 (25.5)	10 (33.3)	0.351	0.685 (0.309–1.521)	—	—
	Grade II	123 (36.5)	8 (26.7)	0.281	1.581 (0.683–3.858)	—	—
	Grade III	62 (18.4)	6 (20.0)	0.829	0.902 (0.354–2.300)	—	—
	Not classified	66 (19.6)	6 (20.0)	0.956	0.974 (0.393–2.479)	—	—

Duration of diagnosis	Recently	156 (46.3)	10 (33.3)	0.172	1.724 (0.783–3.793)	—	—
	Old	181 (53.7)	20 (66.7)	—	—	—	—

Controlled hypertension	Yes	252 (74.8)	27 (90.0)	0.061	0.329 (0.097–1.113)	—	—
	No	85 (25.2)	3 (10.0)	—	—	—	—

Hypertension treatment category	Monotherapy	126 (37.4)	10 (33.3)	0.659	1.194 (0.542–2.633)	—	—
	Bitherapy	129 (38.3)	15 (50.0)	0.208	0.620 (0.293–1.311)	—	—
	Tritherapy	29 (8.6)	2 (6.7)	1	1.318 (0.299–5.815)	—	—
	Quadritherapy	10 (3.0)	2 (6.7)	0.256	0.428 (0.089–2.051)	—	—
	None	43 (12.8)	1 (3.3)	0.234	4.241 (0.563–31.94)	—	—

Tobacco consumption	Yes	10 (3.0)	1 (3.3)	1	0.887 (0.110–7.173)	—	—
	No	327 (97.0)	29 (96.7)	—	—	—	—

Physical activities	Yes	18 (5.3)	5 (16.7)	**0.030**	**0.282 (0.097–0.823)**	0.262	0.505 (0.153–1.924)
	No	319 (94.7)	25 (83.3)	—	—	—	—

Comorbidities	Diabetes	81 (24.0)	9 (30.0)	0.467	0.738 (0.325–1,676)	—	—
	Gout	66 (19.6)	4 (13.3)	0.478	1.583 (0.534–4.689)	—	—
	Heart failure	22 (6.5)	1 (3.3)	0.708	2.025 (0.263–15.573)	—	—
	Eye disease	10 (3.0)	4 (13.3)	**0.020**	**0.199 (0.058–0.678)**	**0.021**	**0.214 (0.058–0.793)**

Weight status (BMI)	Normal	77 (22.8)	9 (30.0)	0.376	0.691 (0.304–1.571)	—	—
	Overweight	109 (32.3)	11 (36.7)	0.629	0.826 (0.380–1.796)	—	—
	Obese	151 (44.8)	10 (33.3)	0.225	1.624 (0.738–3.574)	—	—

*Note*: Variables that were significant in either bivariate or multivariate analysis are highlighted in bold.

**Table 7:** Factors associated with harmful attitudes.

Variables	Terms	Harmful attitudes		*p*	Crude OR (CI 95%)	Adjusted *p*	Adjusted OR (CI 95%)
		Yes, *n* (%)	No, *n* (%)				
Age (years)	<40	11 (5.1)	5 (3.3)	0.399	1.585 (0.539–4.680)	—	—
	40–60	79 (36.7)	53 (34.9)	0.712	1.085 (0.703–1.674)	—	—
	60–80	101 (47.0)	86 (56.6)	0.070	0.680 (0.448–1.033)	—	—
	≥80	24 (11.2)	8 (5.3)	**0.048**	**2.262 (1.987–5.181)**	0.123	1.949 (0.816–4.653)

Sex	Male	86 (40.0)	57 (37.5)	0.629	1.111 (0.725–1.703)	—	—
	Female	129 (60.0)	95 (62.5)	—	—	—	—

Marital status	Married	147 (68.4)	106 (68.7)	0.781	0.938 (0.598–1.471)	—	—
	Single	7 (3.3)	8 (5.3)	0.339	0.606 (0.215–1.708)	—	—
	Divorced	5 (2.3)	3 (2.0)	1	1.183 (0.278–5.025)	—	—
	Widowed	56 (26.0)	35 (23.0)	0.509	1.177 (0.725–1.912)	—	—

Level of education	None	31 (14.4)	18 (11.8)	0.475	1.254 (0.673–2.336)	—	—
	Primary	112 (52.1)	66 (43.4)	0.102	1.417 (0.933–2.151)	—	—
	Secondary	51 (23.7)	37 (24.3)	0.891	0.967 (0.595–1.571)	—	—
	University	21 (9.8)	31 (20.4)	**0.004**	**0.423 (0.232–0.769)**	0.197	0.644 (0.330–1.257)

Monthly income (USD)	Low	128 (59.5)	73 (48.0)	**0.029**	**1.592 (1.047–2.420)**	0.209	1.360 (0.842–2.196)
	Average	70 (32.5)	61 (40.1)	0.250	0.816 (0.462–1.441)	—	—
	High	17 (7.9)	18 (11.8)	0.206	0.639 (0.316–1.285)	—	—

Residence area	Urban	82 (38.1)	74 (48.7)	**0.044**	**0.650 (0.427–0.990)**	0.538	0.852 (0.511–1.420)
	Semi-urban	72 (33.5)	49 (32.2)	0.802	1.058 (0.680–1.648)	—	—
	Rural	61 (28.4)	29 (19.1)	**0.042**	**1.680 (1.017–2.774)**	0.251	1.434 (0.775–2.655)

Occupation	Retailer	41 (19.1)	20 (13.2)	0.134	1.555 (0.870–2.779)	—	—
	Farmer	47 (21.9)	22 (14.5)	0.074	1.653 (0.948–2.882)	—	—
	Unemployed	34 (15.8)	14 (9.2)	0.095	1.852 (0.956–3.585)	—	—
	Teacher	12 (5.6)	16 (10.5)	0.079	0.502 (0.230–1.095)	—	—
	Housewife	29 (13.5)	29 (19.1)	0.148	0.661 (0.377–1.161)	—	—
	Retired	39 (18.1)	36 (23.7)	0.194	0.714 (0.429–1.189)	—	—
	Others	13 (6.0)	15 (9.9)	0.174	0.589 (0.271–1.274)	—	—

Hypertension grade	Grade I	53 (24.7)	43 (28.3)	0.435	0.829 (0.518–1.327)	—	—
	Grade II	82 (38.1)	49 (32.2)	0.245	1.296 (0.837–2.007)	—	—
	Grade III	39 (18.1)	29 (19.1)	0.820	0.940 (0.552–1.601)	—	—
	Not classified	41 (19.1)	31 (20.4)	0.753	0.920 (0.546–1.549)	—	—

Duration of diagnosis	Recently	109 (50.7)	57 (37.5)	**0.012**	**1.714 (1.122–2.617)**	**0.015**	**1.744 (1.112–2.736)**
	Old	106 (49.3)	95 (62.5)	—	—	—	—

Controlled hypertension	Yes	155 (72.1)	124 (81.5)	**0.036**	**0.583 (0.351–0.968)**	0.519	0.816 (0.446–1.503)
	No	60 (27.9)	28 (18.4)	—	—	—	—

Hypertension treatment category	Monotherapy	72 (33.5)	64 (42.1)	0.092	0.592 (0.451–1.063)	—	—
	Bitherapy	89 (41.4)	55 (36.2)	0.314	1.246 (0.812–1.911)	—	—
	Tritherapy	16 (7.4)	15 (9.9)	0.410	0.734 (0.351–1.535)	—	—
	Quadritherapy	5 (2.3)	7 (4.6)	0.247	0.493 (0.154–1.684)	—	—
	None	33 (15.3)	11 (7.2)	**0.018**	**2.324 (1.135–4.760)**	0.128	1.932 (0.828–4.507)

Tobacco consumption	Yes	7 (3.3)	4 (2.6)	1	1.245 (0.358–4.331)	—	—
	No	208 (96.7)	148 (97.4)	—	—	—	—

Physical activities	Yes	14 (6.5)	9 (5.9)	0.818	1.107 (0.466–2.627)	—	—
	No	201 (93.5)	143 (94.1)	—	—	—	—

Comorbidities	Diabetes	57 (28.5)	33 (21.7)	0.292	1.301 (0.797–2.124)	—	—
	Gout	39 (18.1)	31 (20.4)	0.588	0.865 (0.511–1.463)	—	—
	Heart failure	7 (3.3)	16 (105)	**0.005**	**0.286 (0.115–0.714)**	**0.003**	**0.232 (0.088–0.613)**
	Eye disease	6 (2.8)	8 (5.3)	0.272	0.517 (0.176–1.521)	—	—
	Dyslipidemia	9 (4.2)	4 (2.6)	0.427	1.617 (0.489–5.349)	—	—

Weight status (BMI)	Normal	57 (26.5)	29 (19.1)	0.098	1.530 (0.923–2.536)	—	—
	Overweight	76 (35.3)	44 (28.9)	0.198	1.342 (0.957–2.101)	—	—
	Obese	82 (38.1)	79 (52.0)	**0.009**	**0.570 (0.370–0.868)**	**0.035**	**0.620 (0.398–0.968)**

Poor knowledge	Yes	206 (95.8)	131 (86.2)	**0.001**	**3.669 (1.631–8.256)**	**0.003**	**3.500 (1.547–7.920)**
	No	9 (4.2)	21 (13.8)	—	—	—	—

*Note*: Variables that were significant in either bivariate or multivariate analysis are highlighted in bold.

**Table 8:** Factors associated with harmful practices.

Variables	Terms	Harmful practices		*p*	Crude OR (CI 95%)	Adjusted *p*	Adjusted OR (CI 95%)
		Yes, *n* (%)	No, *n* (%)				
Age (years)	<40	8 (2.6)	8 (13.1)	**0.002**	**0.178 (0.064–0.495)**	0.117	0.394 (0.123–1.262)
	40–60	108 (35.3)	24 (39.3)	0.547	0.547 (0.841–1.479)	—	—
	60–80	160 (52.3)	27 (44.3)	0.252	1.380 (0.794–2.399)	—	—
	≥80	30 (9.8)	2 (3.3)	0.099	3.207 (0.746–13.789)	—	—

Sex	Male	124 (40.5)	19 (31.1)	0.170	1.506 (0.837–2.711)	—	—
	Female	182 (59.5)	42 (68.9)	—	—	—	—

Marital status	Married	214 (69.9)	39 (63.9)	0.355	1.312 (0.737–2.336)	—	—
	Single	9 (2.9)	6 (9.8)	**0.024**	**0.278 (0.095–0.812)**	0.133	0.390 (0.114–1.333)
	Divorced	4 (1.3)	4 (6.6)	**0.029**	**0.189 (0.046–0.777)**	0.119	0.294 (0.063–1.369)
	Widowed	79 (25.8)	12 (19.7)	0.310	1.421 (0.719–2.808)	—	—

Level of education	None	43 (14.1)	6 (9.8)	0.377	1.499 (0.608–3.695)	—	—
	Primary	160 (52.3)	18 (29.5)	**0.001**	**2.618 (1.445–4.743)**	**0.042**	**1.316 (1.047–2.676)**
	Secondary	71 (23.2)	17 (27.9)	0.436	0.782 (0.421–1.453)	—	—
	University	32 (10.5)	20 (32.8)	**<0.001**	**0.239 (0.125–0.458)**	**0.028**	**0.701 (0.273–0.986)**

Monthly income (USD)	Low	182 (59.5)	19 (31.1)	**<0.001**	**3.244 (1.802–5.841)**	**0.046**	**1.504 (1.019–3.144)**
	Average	104 (34.0)	27 (44.3)	0.345	0.704 (0.347–1.426)	—	—
	High	20 (6.5)	15 (24.6)	**<0.001**	**0.214 (0.102–0.449)**	0.334	0.714 (0.266–1.917)

Residence area	Urban	123 (40.2)	33 (54.1)	**0.045**	**0.570 (0.328–0.991)**	0.318	0.815 (0.406–1.636)
	Semi-urban	101 (33.0)	20 (32.8)	0.973	1.010 (0.583–1.813)	—	—
	Rural	82 (26.8)	8 (13.1)	**0.023**	**2.425 (1.106–5.319)**	0.438	1.448 (0.569–3.687)

Occupation	Retailer	47 (15.4)	14 (23.0)	0.146	0.609 (0.311–1.194)	—	—
	Farmer	62 (20.3)	7 (11.5)	0.109	1.960 (0.850–4.519)	—	—
	Unemployed	44 (14.4)	4 (6.6)	0.098	2.393 (0.827–6.927)	—	—
	Teacher	16 (5.2)	12 (19.7)	**<0.001**	**0.225 (0.100–0.505)**	0.318	0.743 (0.243–2.273)
	Housewife	54 (17.6)	4 (6.6)	**0.030**	**3.054 (1.063–8.774)**	0.361	1.697 (0.545–5.286)
	Retired	64 (20.8)	11 (18.0)	0.610	1.202 (0.592–2.441)	—	—
	Others	19 (6.2)	9 (14.8)	**0.032**	**0.383 (0,164–0,892)**	0.265	0.571 (0.213–1.530)

Hypertension grade	Grade I	82 (26.8)	14 (23.0)	0.533	1.229 (0.643–2.350)	—	—
	Grade II	103 (33.7)	28 (45.9)	0.068	0.598 (0.343–1.043)	—	—
	Grade III	60 (19.6)	8 (13.1)	0.233	1.616 (0.730–3.579)	—	—
	Not classified	61 (19.9)	11 (18.0)	0.733	1.132 (0.556–2.303)	—	—

Duration of diagnosis	Recently	135 (44.1)	31 (50.8)	0.337	0.764 (0.441–1.325)	—	—
	Old	171 (55.9)	30 (49.2)	—	—	—	—

Controlled hypertension	Yes	230 (75.2)	49 (80.3)	0.388	0.410 (0.375–1.467)	—	—
	No	76 (24.8)	12 (19.7)	—	—	—	—

Hypertension treatment category	Monotherapy	110 (35.9)	26 (42.6)	0.324	0.755 (0.432–1.321)	—	—
	Bitherapy	119 (38.9)	25 (41.0)	0.760	0.916 (0.524–1.604)	—	—
	Tritherapy	28 (9.2)	3 (4.9)	0.278	1.947 (0.573–6.621)	—	—
	Quadritherapy	9 (2.9)	3 (4.9)	0.429	0.586 (0.154–2.230)	—	—
	None	40 (13.1)	4 (6.6)	0.153	2.143 (0.737–6.228)	—	—

Poor knowledge	Yes	287 (93.8)	50 (82.0)	**0.008**	**3.323 (1.492–7.403)**	**0.015**	**2.766 (1.218–6.281)**
	No	19 (6.2)	11 (18.0)	—	—	—	—

Harmful attitudes	Yes	189 (61.8)	26 (42.6)	**0.006**	**2.175 (1.245–3.797)**	**0.022**	**1.946 (1.100–3.443)**
	No	117 (38.2)	35 (57.4)	—	—	—	—

Tobacco consumption	Yes	9 (2.9)	2 (3.3)	1	0.894 (0.188–4.243)	—	—
	No	297 (97.1)	59 (96.7)	—	—	—	—

Physical activities	Yes	14 (4.6)	9 (14.8)	**0.007**	**0.277 (0.114–0.673)**	0.298	0.581 (0.209–1.615)
	No	292 (95.4)	52 (85.2)	—	—	—	—

Comorbidities	Diabetes	80 (26.1)	10 (16.4)	0.106	1.805 (0.875–3.725)	—	—
	Gout	81 (19.9)	9 (14.8)	0.347	1.439 (0.672–3.080)	—	—
	Heart failure	20 (6.5)	3 (4.9)	0.779	1.352 (0.389–4.899)	—	—
	Eye disease	11 (3.6)	3 (4.9)	0.712	0.721 (0.195–2.865)	—	—
	Dyslipidemia	11 (3.6)	2 (3.3)	1	1.100 (0.238–5.092)	—	—

Weight status (BMI)	Normal	75 (24.5)	11 (18.0)	0.275	1.476 (0.731–2.980)	—	—
	Overweight	102 (33.3)	18 (29.5)	0.561	1.194 (0.858–2.175)	—	—
	Obese	129 (42.2)	32 (52.5)	0.139	0.660 (0.381–1.146)	—	—

*Note*: Variables that were significant in either bivariate or multivariate analysis are highlighted in bold.

## Data Availability

The data that support the findings of this study are available on request from the corresponding author. The data are not publicly available due to privacy or ethical restrictions.

## References

[1] Kreutza R., Brunströmb M., Burnierc M. (2024). European Society of Hypertension Clinical Practice Guidelines for the Management of Arterial Hypertension. *European Journal of Internal Medicine*.

[2] Yaya H. S., Kengne A. P. (2014). *Le Défi de la Prévention des Maladies Cardiovasculaires et ses Perspectives en Afrique: Juguler le mal Meurtrier et Insidieux de l’Hypertension Artérielle*.

[3] N. C. D. Risk Factor Collaboration (NCD-RisC) (2021). Worldwide Trends in Hypertension Prevalence and Progress in Treatment and Control From 1990 to 2019: A Pooled Analysis of 1201 Population-Representative Studies With 104 Million Participants. *Lancet (London, England)*.

[4] Boateng E. B., Ampofo A. G. (2023). A Glimpse into the Future: Modelling Global Prevalence of Hypertension. *BMC Public Health*.

[5] Ferdinand K. C. (2020). Uncontrolled Hypertension in Sub-Saharan Africa: Now is the Time to Address a Looming Crisis. *The Journal of Clinical Hypertension*.

[6] Touré M., Ibrahima S., Camara Y. (2024). Screening, Treatment and Control of High Blood Pressure on Five Sites in Mali. *World Journal of Cardiovascular Diseases*.

[7] Simeni Njonnou S. R., Eyenga Bangbang C. F., Christian N. O. (2024). Prevalence and Determinants of Hypertension in a Semi-Urban Population: A Cross-Sectional Study in Dschang (West Region of Cameroon). *The Pan African medical journal*.

[8] Kuate Defo B., Mbanya J. C., Kingue S. (2019). Blood Pressure and Burden of Hypertension in Cameroon, a Microcosm of Africa: A Systematic Review and Meta-Analysis of Population-Based Studies. *Journal of Hypertension*.

[9] Wierzejska E., Giernaś B., Lipiak A., Karasiewicz M., Cofta M., Staszewski R. (2020). A Global Perspective on the Costs of Hypertension: A Systematic Review. *Archives of Medical Science*.

[10] Yu E. Y. T., Wan E. Y. F., Mak I. L. (2023). Assessment of Hypertension Complications and Health Service Use 5 Years After Implementation of a Multicomponent Intervention. *JAMA Network Open*.

[11] Li Y., Yuan X., Zheng Q. (2023). The Association of Periodontal Disease and Oral Health With Hypertension, NHANES 2009–2018. *BMC Public Health*.

[12] Pietropaoli D., Del Pinto R., Ferri C. (2018). Poor Oral Health and Blood Pressure Control Among US Hypertensive Adults. *Hypertension. American Heart Association*.

[13] Tonetti M. S., Greenwell H., Kornman K. S. (2018). Staging and Grading of Periodontitis: Framework and Proposal of a New Classification and Case Definition. *Journal of Periodontology*.

[14] Belinga L. E. E., Ngan W. B., Mouliom J. S. K., Choukem S. P. (2013). Evaluation de la Santé Bucco-Dentaire des Patients Diabétiques Camerounais. *Health Sciences and Disease*.

[15] Kamagate A., Coulibaly N. T., Kone D., Brou E., Bakayoko Ly R. (2001). Les Parodontites en Afrique Noire. Influences des Facteurs Socio-Économiques et Habitudes Culturelles. *Odonto-Stomatologie Tropicale*.

[16] Rasoariseheno F., Raveloson F., Rakotoarivony A., Andrianavony N. R., Rabearivony N. (2012). Relation Entre Maladies Parodontales et Maladies Cardiovasculaires à Madagascar. *Revue d’odontostomatologie Malgache. Madagascar*.

[17] Essama Eno Belinga L., Dakam W., Ndjoh J. (2020). Association Entre Obesité et Parodontite: Étude Transversale A L’hôpital Général de Douala (Cameroun).

[18] Essama E. B. L., Sidick M. A., Abdoulaziz D. (2020). Santé Parodontale et Hypertension Artérielle: Étude Pilote à l’Hôpital Général de Douala. *Revue de Médecine et de Pharmacie*.

[19] Eno Belinga L. E., Bell Ngan W., Lemougoum D. (2018). Association Between Periodontal Diseases and Cardiovascular Diseases in Cameroon. *Journal of Public Health in Africa*.

[20] Leye M., Diouf M., Térence Madozein W. S. (2014). Hypertension and Periodontal Status in Senegalese Patients: A Case-Control Study. *Open Journal of Epidemiology*.

[21] Simeni Njonnou S. R., Boombhi J., Etoga M. C. E. (2020). Prevalence of Diabetes and Associated Risk Factors among a Group of Prisoners in the Yaoundé Central Prison. *Journal of Diabetes Research*.

[22] Essi M.-J., Njoya O. (2013). L’enquête CAP (Connaissances, Attitudes et Pratiques) en Recherche Médicale. *Health Sciences and Disease*.

[23] Kaguru G., Ayah R., Mutave R., Mugambi C. (2022). Integrating Oral Health Into Primary Health Care: A Systematic Review of Oral Health Training in Sub-Saharan Africa. *Journal of Multidisciplinary Healthcare*.

[24] Rasouli-Ghahroudi A. A., Khorsand A., Yaghobee S. (2016). Oral Health Status, Knowledge, Attitude and Practice of Patients With Heart Disease. *ARYA Atherosclerosis*.

[25] Sanchez P., Everett B., Salamonson Y. (2019). The Oral Health Status, Behaviours and Knowledge of Patients With Cardiovascular Disease in Sydney Australia: A Cross-Sectional Survey. *BMC Oral Health*.

[26] Saud A. A., Saud A. N., Alnufaiy Banna M. (2020). Assessment of Dental Health Status, Knowledge, and Practice Among Saudi Diabetic Patients Attending General Practice Clinic. *Journal of Nature and Science of Medicine*.

[27] Berthé D. (2020). Motifs De Consultation Des Patients Dans le Service d’Odontostomatologie De l’Hopital De Kayes en 2018, Mali. *Mali Santé Publique*.

[28] Gavaza P., Rogers T., Mosavin R. (2017). California Dentists’ Opinions of the Interface Between Oral and Overall Health. *Journal of the California Dental Association*.

[29] Kane Aboubacar S. T., Guirassy Mouhamadou L., Kadidia T. (2020). Évaluation de l’hygiène Buccodentaire des Patients Consultant le Service D’odontostomatologie du Centre De Santé De Référence De Ouelessebougou Au Mali. *African Journal of Dentistry & Implantology*.

[30] Okeigbemen S. A., Awhoregba T. O., Ojuola G. T. (2021). Oral Health-Related Knowledge, Attitude and Practices Among Trainee Community Health Officers in A Nigerian Tertiary Health Institution. *African Journal of Biomedical Research*.

[31] Mishra A., Sharma D., Tripathi G. M., Khan T. A. (2023). Rural-Urban Disparities in Knowledge, Attitude, and Practice Toward Child Oral Health Among Mothers of 9-36-Month-Old Children. *Journal of Rural Medicine*.

[32] Kim C. M., Lee S., Hwang W. (2022). Obesity and Periodontitis: A Systematic Review and Updated Meta-Analysis. *Frontiers in Endocrinology*.

[33] Cervantes F., López-Garrido P., Montero M. I. (2010). Early Intervention During Imatinib Therapy in Patients With Newly Diagnosed Chronic-Phase Chronic Myeloid Leukemia: A Study of the Spanish PETHEMA Group. *Haematologica*.

[34] Aliyu T. K., Titus O. S., Bernard O. T., Alade O. T., Ehizele A. O., Foláyan M. O. (2025). Cultural Themes Related to Oral Health Practices, Beliefs, and Experiences in Nigeria: A Scoping Review. *Oral*.

[35] Bethesda (2021). Effect of Oral Health on the Community, Overall Well-Being, and the Economy. *Oral Health in America: Advances and Challenges*.

[36] Miezah D., Hayman L. L. (2024). Culturally Tailored Lifestyle Modification Strategies for Hypertension Management: A Narrative Review. *American Journal of Lifestyle Medicine*.

